# Supplementary material for: Are carbon emissions trading and green financial instruments synergistic? -Comprehensive quantitative research based on content analysis
Source: PLoS One. 2024 Mar 7;19(3):e0298601. doi: 10.1371/journal.pone.0298601 (PMC10919601; doi:10.1371/journal.pone.0298601)
Supplement: S1 Data — (ZIP) [file pone.0298601.s001.zip › Data/地级市Z_F工作报告.zip!/╔┬╬≈/╤╙░▓╩╨/└┤╫╘╣1⁄2╓┌║┼í╛┬φ┐╦╩2╛▌═°í┐_2017.docx]

各位代表：

现在，我代表市人民政府向大会报告工作，请予审议，并请市政协委员和其他列席人员提出意见。

一、2016年和第四届政府工作回顾

2016年，是我市进入新世纪以来发展最为艰难的一年。我们顶着经济下行压力迎难而上，扭住转型发展毫不松懈，聚焦精准脱贫合力攻坚，全力稳增长、促投资、调结构、抓改革、惠民生，经济总体呈现缓中趋稳、稳中有进态势。尽管全市上下同心同德，苦干实干，但在油煤量价齐跌、新动能还没有完全显现的情况下，四项经济指标未能实现预期增长，其他各项省考指标均完成或超额完成任务。全年完成生产总值1082.9亿元，增长1.3%；全社会固定资产投资1359.3亿元，下降15.4%；地方财政收入130.5亿元，下降19%；城镇居民人均可支配收入30693元，增长7.4%；农村居民人均可支配收入10568元，增长8%。

全力保持经济稳定增长。制定推进供给侧结构性改革、打好“三去一降一补”攻坚战和降低实体经济企业成本、工业稳增长调结构促投资、防范市本级财政金融风险、房地产去库存等一系列政策措施，引导退出小煤矿6家、淘汰落后产能150万吨，房地产库存面积环比连降10个月。生产原油1514.3万吨、下降9.6%，加工原油927.5万吨、下降8.1%，生产煤炭4381.5万吨、下降0.4%，天然气33.7亿立方米、下降1.2%，卷烟36.9万箱、下降7.1%，LNG 产量76.7万吨、增长45.1%，发电38.5亿千瓦时、增长5.7倍。第一产业增加值增速居全省第一。生产粮食78万吨，增长8%。苹果产量303.2万吨，增长10.9%。生产蔬菜129.9万吨，增长7.4%。猪牛羊饲养量和肉蛋奶产量稳定增长。实现社会消费品零售总额256.5亿元，增长6.4%。

加快经济结构调整步伐。启动八大产业集群和五大文化旅游产业板块建设，延安综合能源基地发展规划正式纳入国家能源规划盘子，组建了陕西延安电业公司，永坪20万吨航空煤油、志丹轻烃综合加工和一批新能源发电项目建成投产，延安煤油气资源综合利用、大唐热电联产、华能延安电厂等项目建设步伐加快，华为云计算数据中心、彩虹光伏玻璃等项目开工建设，北京云真视讯、英雄互娱、898创新空间、一正启源等一批成长性好的“瞪羚企业”落户延安，新“延安”牌卷烟投放市场，多业并举、多元发展、多极增长的格局正在形成。县域工业集中区新入驻企业20户，营业收入增长10.2%。明确把“一村一品、一乡一业”作为乡镇工作主线，建成了一批国家级和省级示范县、示范镇、示范村。新启动13个现代农业示范园区和8个农产品加工园区建设，成功举办第一届世界苹果大会，“洛川苹果”荣获2016年全国果蔬产业十大最具影响力地标品牌，甘泉豆腐、甘泉黄酒、吴起荞麦香醋、黄龙蜂蜜被批准为国家地理标志保护产品，富县直罗贡米获得国家地理标志产品登记。圣地河谷金延安一期、延安北京知青博物馆等文化旅游项目建成，黄帝文化、黄河文化、黄土风情文化等六大文化产业园区建设取得新进展，“绿色黄龙”成为省际旅游新亮点。成功举办第十一届中国艺术节开幕式。来延游客突破4000万人次，旅游综合收入228亿元。红色教育培训33.4万人次。新增市场主体2.6万户、私营企业4903户，新入库“四上”企业169户。第三产业、非公经济占GDP比重分别达到36.1%和26.1%，分别提高5.8和2.7个百分点。

狠抓重大基础设施项目建设。市级重点项目开复工率达到95%，较上年提高7.2个百分点。出台政府和社会资本合作项目管理办法，13个PPP项目开工建设或取得实质性进展。争取国家专项建设基金41亿元、项目65个。延安新机场完成飞行区土方工程。黄延高速扩能工程建成通车，蒙华铁路、吴定高速等项目进展顺利，西延高铁和绥德至延川、清涧至子长高速公路开工建设。南沟门水利枢纽工程全面建成，黄河引水工程黄延线全线贯通。延河综合治理城区段初见成效。完成造林76.7万亩，治沟造地8.2万亩。

全面推进城镇体系建设。新区初具城市形象，为民服务中心搬迁入驻，延安大剧院投入运营，学习书院、3所幼儿园、桥沟新老城连接线和21条市政道路建成，部分居民安置房、保障房和干部职工住房具备交房条件，水电路讯、公园广场、综合管廊、运动休闲场所等公共服务设施加快推进。老城改造步伐加快，2.39万套棚户区改造项目全部开工，南门广场、火车站西广场建成开放，延河两岸城市夜间文化旅游提升工程成为旅游新热点。城区空气质量优良天数达到290天，“圣地蓝”成为一张靓丽名片。一批突出环境保护问题整改到位。“2+1”三城联创首战告捷，荣获“国家森林城市”称号，国家卫生城市创建通过省级综合鉴定和国家暗访考评，园林城市创建通过国家技术专家组评估，创建全国文明城市、国家节水型城市和国家环保模范城市工作扎实推进。被列为第三批国家新型城镇化综合试点地区。安塞实现撤县设区。县城、重点镇、新型农村社区承载服务功能稳步提升，城镇化率达到59.09%，提高1.77个百分点。

深入推进改革开放。完成年度改革任务133项。首批6户市属国企改制顺利完成，剩余40户全部启动。清理行政审批事项47项，全面推行网上审批、限时办结、重大项目代办、集中办证等制度，群众对简政放权满意度达到98.8%。成立西北五省唯一一家地市级保险监管分局，设立了总规模150亿元的延安光大城市发展基金和陕西金控延安融资担保公司。银行存款余额1452.5亿元，贷款余额927.5亿元，存贷比63.9%。全面推开“营改增”试点。子长县“平价医院”模式荣获国家“地方政府改革与创新奖”。机关事业单位养老保险制度改革、水电气和污水处理价格改革顺利推进。流转农村承包耕地69.6万亩。镇村综合改革任务全面完成，撤并行政村1614个。实施招商项目495个，到位资金705.6亿元，增长13.8%。

持续保障和改善民生。启动3.22万户、7.76万贫困人口易地扶贫搬迁工程，4.86万无劳动能力贫困人口全部纳入兜底保障，9.01万贫困人口和宝塔、安塞、子长、甘泉4县区实现脱贫，538个贫困村脱贫退出，全市贫困发生率由6.4%下降到0.76%。

新增城镇就业1.63万人，城镇登记失业率3.55%。提高城乡低保标准和居民基本医疗保险补贴标准，13.7万城乡低保对象实现应保尽保。新建改建医院7所、卫生院16所。推进城市公立医院医疗服务价格改革，实现药品零差率销售。延安医疗集团完成对市第二人民医院托管。新建改建各类学校178所。全面实施十五年免费教育。延安大学新校区开工建设。成功举办全国2016年全民健身和第十八届中国科协年会科普活动启动仪式。加强社会治理创新，来市赴省信访分别下降15.8%和40.1%，化解信访积案410件。安全生产形势总体平稳，平安延安建设取得新成效，群众社会治安满意度达到88.12%，社会大局保持和谐稳定。

各位代表，2016年各项工作的较好收尾，为第四届政府五年奋斗历程划上了圆满句号。过去五年，我们在省委、省政府和市委的正确领导下，认真实施市第四次党代会确定的“四大战略”，坚持以统筹城乡发展总揽工作全局，主动适应和引领经济发展新常态，有效应对经济下行压力，积极克服特大自然灾害影响，攻坚克难，扎实苦干，较好地完成了市四届人大一次会议确定的主要目标任务。

这五年，综合实力跃上新台阶。经济总量、固定资产投资先后突破千亿元大关，累计完成生产总值6292.4亿元、全社会固定资产投资6890.7亿元，分别是前五年的1.5倍和2.4倍。五年完成地方财政收入754.5亿元，是前五年的1.6倍。城乡居民人均可支配收入分别较2011年提高9505元和4003元。人均GDP超过全国全省平均水平。人均财力位居全省前列，年均向中省财政贡献166亿元。粮食面积和产量稳定在300万亩和70万吨以上。

这五年，结构调整实现新突破。整合组建延安高新技术产业开发区，“三区九园”工业框架布局基本成型，17个县域工业集中区入驻企业394户，累计实施结构调整项目239个，183个重大能源转化和非能源项目建成投产。被国家命名为现代农业示范区，启动建设现代农业示范园区100个，累计建成50个，洛川国家级苹果批发市场一期建成投用。苹果面积、产量居全国地级市之首，洛川苹果、延川红枣、黄龙核桃、吴起“胜利山”醋被认定为中国驰名商标，特色农产品品牌达到120多个。建成西北局纪念馆、枣园文化广场等一批重大文化旅游项目，接待游客和旅游综合收入分别较2011年增长96.4%和107.3%。孵化培育电商企业750多家，与阿里巴巴等知名电商企业建立了密切合作关系。五年新增市场主体8.02万户，入库“四上”企业1031户。第三产业、非公经济占GDP比重较2011年分别提高17.1和8个百分点。三次产业结构由2011年的7.8：73.2：19调整为10.9：53：36.1。

这五年，城乡统筹取得新进展。新区岩土工程堪称世界一大创举，四年多完成各类建设项目110多个，建设速度之快被誉为延安的“深圳速度”，开创了延安城市建设新纪元。城市改造让延安老城旧貌换新颜，实施棚户区改造项目59个，12万山体居民下山安居，中疏外扩目标正在实现。31个重点镇基本成型，56个新型农村社区基本达标，29万农民进城落户，城镇化率五年提高9.62个百分点。“2+1”三城联创带动城市管理水平全面提升。率先在全国实施新一轮退耕还林160.2万亩，五年新造林364万亩，治沟造地36.7万亩。单位GDP能耗下降16%，节能减排任务全面完成。城区空气质量优良天数稳居全省前列。被列为全国生态文明先行示范区和第二批低碳城市试点。开通延安至西安、宝鸡动车。新增延安至广州、上海、重庆等4条城市航线。4条高速公路建成通车，高速公路通车里程突破800公里。水利、电力、通信保障能力明显提升。

这五年，改革开放达到新水平。启动实施改革事项419项，完成中省改革任务381项。完成4家农商行改制，交通、光大、长安、北京、西安、重庆6家银行在延设立分支机构，银行存贷款余额突破2300亿元。设立3个院士工作站。完成农村土地承包经营权确权登记颁证、集体林权制度改革、公车改革和食药、工商、质监、盐务等管理体制改革。海关和出入境检验检疫机构正式运行，与重点口岸建立通关协作机制。累计完成外贸进出口总额26.3亿元，年均增长31.5%。五年招商引资到位资金突破2000亿元，华为集团、大唐集团等世界500强企业落户延安。

这五年，人民生活有了新提升。累计民生投入1212亿元，是前五年的2倍。43.7万贫困人口和吴起、志丹、黄龙7个县区整体脱贫。基本消除零就业家庭。率先在全省实现城乡居民医疗保险、养老保险制度一体化，城市公立医院改革被国家确定为试点。实现学前三年免费教育、义务教育营养改善计划全覆盖。新建和改扩建幼儿园158所、中小学校166所，5个县区实现省级教育强县目标，9个县区实现“双高双普”，9个县区通过国家义务教育发展基本均衡县评估认定。文化、科技、体育等社会事业蓬勃发展。保障房建设走在全省前列，10.7万户群众住房困难得到解决。取得抗洪抢险和灾后重建全面胜利，7.4万因灾房屋倒损户搬入新居。广泛开展“延安模范”评树活动。社会治理不断创新，“两说一联”经验在全国推广。荣获全国法治宣传教育先进市和全国社会治安综合治理优秀市称号，实现全国双拥模范城“八连冠”。

这五年，作风建设取得新成效。扎实开展党的群众路线教育实践活动和“三严三实”专题教育、“两学一做”学习教育，政风行风持续好转。政府部门权力和责任两张清单改革走在全省前列，累计精简下放审批事项201项，精简率53.9%。“三公”经费持续下降。实行国有资产统一管理，公共资源交易中心投入运行。建立事业单位逢进必考和临聘人员管理制度。坚持开展公民代表走进市政府和大学生到政府机关见习活动。网民留言和市民热线办结率达到100%。坚决纠正侵害群众利益的不正之风，严肃查处腐败问题。办理人大代表建议580件、政协委员提案1948件。

监察、物价、统计、文物、气象、税务、方志、档案、慈善、老龄、助残、民族宗教、妇女儿童、外事侨务、国家安全、人民防空、国防动员、驻延部队和民兵预备役建设等工作都取得了新成绩。

各位代表，回首过去的五年，困难比预料的多，结果比预想的好，成绩来之不易。这是市委统揽全局、正确领导的结果，是市人大、市政协依法监督、大力支持的结果，更是全市人民同心协力、团结拼搏的结果。在此，我代表市人民政府，向全市广大干部群众，向各民主党派、工商联、无党派人士和离退休老干部，向中省驻延企事业单位、驻延部队、武警官兵和公安政法干警，向各位人大代表、政协委员和所有关心、支持延安建设发展的社会各界人士表示崇高的敬意和衷心的感谢！

在肯定成绩的同时，我们也清醒地认识到，延安仍属欠发达地区，发展中存在许多突出矛盾和问题：一是经济结构单一的局面没有根本改变，非公经济发展活力不足，传统发展优势正在减弱，新的增长动能尚未形成，经济增速连续八年全省垫底，发展不优、发展不快是最大实际；二是城乡发展差距大，县域发展不平衡，基础设施欠账多，公共服务均等化水平不高，推动城乡一体发展是最大挑战；三是资源环境刚性约束趋紧，节能减排任务更加艰巨，生态环境脆弱是最大制约；四是深层次社会矛盾日渐凸显，新老问题交织叠加，维护社会稳定是最大压力；五是一些部门依法行政意识不强、行政效能不高，一些干部不重视学习、不研究工作，不作为、慢作为、乱作为现象依然存在，发展环境不优是最大羁绊。针对这些问题，我们将不回避、不护短，认真研究，对症下药，切实加以解决。

二、新一届政府工作的总体思路和目标任务

今后五年是我市加快转型发展、奋力追赶超越的关键期，是率先实现精准脱贫、全面建成小康社会的决胜期。习近平总书记指出“陕西正处于追赶超越阶段”，延安具有“两个明显优势、一个明显制约”，为我们未来发展指明了前进方向；国家继续实施“一带一路”和西部大开发战略，给革命老区“量身定做”了诸多利好政策，为我们加快发展创造了有利条件；深入推进供给侧结构性改革，扩大有效需求，振兴实体经济，为我们推动结构调整、实现转型升级带来了新的机遇；省委、省政府始终对延安厚爱有加，全方位倾斜支持，为我们增添了追赶超越的信心和决心；市第五次党代会描绘了今后五年发展的宏伟蓝图，进一步激发了干部群众干事创业的热情和干劲，全市上下已经形成了人心思进、你追我赶的良好氛围。只要我们坚持问题导向，敢于直面矛盾，趋利避害，扬优成势，低调务实，埋头苦干，就一定能够再创延安发展新的辉煌！

按照市第五次党代会和全市“十三五”发展规划部署，今后五年工作的总体思路是：高举中国特色社会主义伟大旗帜，认真贯彻党的十八大和十八届三中、四中、五中、六中全会精神，深入贯彻习近平总书记系列重要讲话特别是回陕、回延重要讲话精神，统筹推进“五位一体”总体布局，协调推进“四个全面”战略布局，深入践行“五大发展理念”，全面落实“五个扎实”要求，坚持用延安精神建设延安，大力实施“高端能化强市，特色产业富民，文化旅游带动，生态环境提升，城乡一体发展”战略，加快转型发展，奋力追赶超越，推动改革创新，增进民生福祉，决战决胜全面小康，努力让延安人民生活得更幸福更美好。

通过五年坚持不懈地努力，扎实走好改革发展新的长征路，力争实现“一个率先、两个追赶、三个超越”目标，使延安的经济实力更强、民生福祉更多、文明程度更高、生态环境更美、政治生态更好，让老人们都愿意在这里养老，孩子们都乐意在这里上学，年轻人都喜欢在这里创业，投资者都竞相在这里发展，让来延旅游的人流连忘返，让革命圣地延安真正成为全国人民心目中向往的、神圣的地方。

“一个率先”，即率先在全国革命老区中实现整体脱贫。把精准脱贫作为最大政治任务，以“两区一带”为主战场，因地制宜，精准发力，确保2018年贫困户全部脱贫、贫困村全部退出、贫困县全部摘帽。

“两个追赶”，即生产总值、地方财政收入增速追赶全省平均水平。生产总值和地方财政收入年均分别增长7%左右，人均生产总值、人均财力稳居全省第一方阵。

“三个超越”，即全社会固定资产投资增速、城镇居民和农村居民人均可支配收入水平超越全国平均水平。全社会固定资产投资年均增长15%，城镇居民人均可支配收入年均增长10%，农村居民人均可支配收入年均增长12%。

实现五年奋斗目标，我们必须牢牢把握以下几点：必须把转型发展作为第一要务，以新理念引领新发展，坚定不移调结构、促转型，努力实现有质量、有效益、可持续的发展；必须把追赶超越摆在第一位置，对照标杆，奋起直追，正视差距，补齐短板，发挥优势，应时而动，努力实现争先进位；必须把改革开放作为第一动力，解放思想，大胆创新，破解难题，突破体制机制束缚，释放发展活力，激活内生动力；必须把人民需求作为第一取向，切实维护群众利益、维护公平正义、维护社会稳定，把人民对幸福生活的憧憬变成我们的实际行动；必须把狠抓落实作为第一职责，建立“落实型”政府，“实”字当头，“干”字为先，扑下身子真干、苦干、实干，持之以恒落细、落快、落实，以政府的敢担当、敢作为，努力赢得人民群众的信赖和支持。

重点抓好五个方面工作：

（一）加快推进高端能化，力促工业转型升级。坚持把优化升级作为主方向、园区作为主战场、项目作为总抓手、品牌作为助推器，全面加快陕北高端能化基地建设，推动能源初级产品深度转化，改造提升传统产业，培育壮大新兴产业，调整优化工业内部结构。以“三区九园”、延安高新区、县域工业集中区为载体，完善配套服务功能，提高产业孵化水平，增强产业承载、要素集聚能力，推动园区经济体量、承载容量、发展质量大幅提升。加快八大产业集群建设，大力实施《延安综合能源基地发展规划》，建设石油、煤炭、天然气和煤电“四大基地”，推进油煤气资源综合转化利用、战略性新兴产业和非能产业项目建设，推动能源由量的扩张向质的提升跨越，能化由初级加工向产业链中高端跨越，提升高端能化对经济总量和财政收入的贡献率。

（二）加快发展特色产业，夯实群众脱贫致富基础。以提升国家现代农业示范区建设水平为目标，加快苹果、蔬菜、小杂粮等优质有机农产品生产加工基地建设，抓好200个现代农业示范园区和8个农产品加工园区建设。推进“一村一品、一乡一业”，因地制宜发展林果业、养殖业和设施农业，培育新型经营主体，引导更多农民从事农产品加工、流通和营销，促进农业增效、农民增收。

加强农产品质量安全监测、农业生产技术服务和防灾减灾体系建设，扩大苹果、红枣、小杂粮、地椒羊等特色农产品品牌影响力，提升农业产业化水平。实施“六个一批”脱贫工程，确保2018年实现率先脱贫目标，不让一户群众在全面小康路上掉队。

（三）加快构筑全域旅游新格局，带动提升现代服务业发展水平。全力打造红色旅游、黄帝历史文化旅游、黄河自然遗产旅游、黄土风情文化旅游、自然绿色生态旅游等五大文化旅游产业板块，狠抓文化资源挖掘、景区开发提升、基础设施配套和服务功能完善，构建全景式覆盖、全产业发展、全季节体验、全社会参与的全域旅游新格局，推动文化旅游资源优势向经济优势转变。推进六大文化产业园区建设、革命旧址保护提升、南泥湾景区开发、历史文物和大遗址保护等重大文化旅游项目，建成一批文化旅游名镇、文化古镇、特色旅游示范村。做大做强红色教育培训产业。加快文化产业开发，提升文化产业增加值占比。大力发展电子商务、现代物流、中介咨询、健康医养、文化创意、研学培训等新型服务业态，培育新的经济增长点。

（四）加快生态文明建设，增强可持续发展能力。坚持绿色发展理念，全面实施主体功能区规划。加速推进“陕北高原大绿化”，筑牢黄土高原生态屏障，森林覆盖率达到50%。完成国有林场改革。实行最严格的水资源管理制度，完成延河综合治理。推进城乡环境综合整治，全面改善人居环境。大力发展循环经济，推动资源集约节约利用，单位GDP能耗累计下降16%。创新生态保护机制，编制产业准入负面清单，深化自然资源资产负债表编制试点和领导干部自然资源资产离任审计试点工作。建成全国生态文明先行示范区。

（五）加快城乡一体化进程，实现城乡群众共享发展。认真开展国家新型城镇化综合试点，推动城乡统筹、产城融合和城市绿色智能发展。优化城镇空间布局，积极推进子长、黄陵撤县设市和甘泉撤县设区，构建“一主两副”城市格局和“一城三区”中心城市圈。加快完善新区公共服务设施和产业配套，打造城市经济增长新引擎。坚持历史风貌和现代特色并重，开展城市修补和生态修复。创新城市管理体制，提升城市管理服务水平，建设宜居宜业宜游现代城市，全面实现“2+1”三城联创目标。增强县城、重点镇承载服务功能，促进公共服务向农村延伸、人口向城镇聚集，城镇化率提高到65%以上。坚持互联互通、城乡共享原则，持续改善交通、水利、电力、信息等基础设施条件。推进公共服务均等覆盖，积极回应群众关切，让人民群众享有更稳定的就业、更满意的教育、更优质的医疗、更舒适的住房、更便捷的交通，让老百姓有更多获得感。创新社会治理，保障社会公平正义，维护社会大局和谐稳定，增强人民群众安全感。

三、2017年重点工作任务

今年是贯彻落实市第五次党代会精神的开局之年。全市经济工作的总体思路是：坚持稳中求进工作总基调，紧扣追赶超越定位，以提高发展质量和效益为中心，以推进供给侧结构性改革为主线，强力推进“转型升级十大产业项目”，加快实施“基础设施十大建设工程”，认真办好“人民幸福十件实事”，推动经济社会平稳健康发展。主要预期目标是：生产总值增长7%，全社会固定资产投资增长15%，地方财政收入增长5%，城乡居民人均可支配收入分别增长9%和10%。以上目标的确定，既是完成“十三五”规划任务、实现全面小康社会的需要，也是加快转型发展、奋力追赶超越的需要。无论面临多大压力，我们都必须坚定发展信心不动摇，保持凝心聚力干事创业的精气神，大力弘扬延安精神，开拓创新，埋头苦干，以发展的新成效回应人民群众的新期待。

重点抓好六个方面工作：

（一）依托三大特色资源优势，着力在推动产业转型升级上下功夫

把2017年作为转型发展的攻坚年，突出抓好“转型升级十大产业项目”，推动产业优化升级，打造延安经济升级版。

全力推进高端能化产业发展。积极为能源企业创造良好生产环境，支持企业释放先进产能，稳定主要工业品产量。生产原油1573万吨，加工原油985万吨，生产煤炭5100万吨，生产天然气45亿立方米，LNG产量100万吨。出台加快产业园区建设的意见，完善配套服务设施，提升园区承载项目、聚合产业、服务企业能力。加快延长石油集团延安煤油气资源综合利用、洛川轻烃裂解综合利用、延长石油伴生气资源循环利用和华能延安电厂等项目建设，做好店头电厂、富县电厂和子长电厂开工前期工作。建成大唐热电联产和吴起、延川、宜川、黄龙等县区19个风电、光伏新能源项目，全年发电40亿千瓦时。加快子长、志丹两个天然气净化厂、宝塔50万吨环保型煤加工、甘泉混合烃改扩建等项目建设，推进子长500万吨煤炭热解气化一体化综合利用项目，建成子长100万吨中低温热解制取清洁燃料和志丹、安塞各20万吨天然气液化等项目。上半年开工建设新区和安塞多能互补集成优化示范工程。

促进现代农业提质增效。深入推进农业供给侧结构性改革，扩大绿色优质农产品有效供给。大力发展“一村一品、一乡一业”，重点扶持16个专业乡镇、210个专业村做大做强，形成特色化竞争、差异化发展格局。实施苹果产业转型升级工程，新增苹果面积11万亩，其中发展矮密栽培5万亩。支持特色养殖、绿色蔬菜、花卉苗木产业发展，新建和改造设施蔬菜1万亩。加快推进安塞省级肉羊产业转型升级示范区和3个县区省级肉羊基地县建设，新建60个规模化养殖场或养殖小区。抓好优质小杂粮种植基地建设。新启动现代农业示范园区20个，年内建成10个。推进甘泉省级农产品加工产业示范园区建设。加快农村一二三产融合发展，壮大农民专业合作社、种养大户和家庭农场，每个县区至少引进或培育1家国内外知名农业龙头企业，参与果菜畜粮等优质农产品深加工和仓储、冷链配送，开发营养保健品、休闲食品、航空食品，打造休闲农庄、精品民宿、美丽田园等新业态。推行农业标准化生产和“三品一标”认证，建立农产品质量安全追溯体系，严把从农田到餐桌的每一道防线。大力发展电子商务和订单直销，打造线上线下相融合的农产品营销平台。加强特色农产品品牌培育和管理保护，以品牌效应扩大市场占有率，促进农民持续增收。

提升文化旅游产业发展水平。大力发展“旅游+”，策划启动一批有特色、有吸引力的重大文化旅游项目，推进旅游园区化、项目化、实体化，加快打造延安大景区。引进有实力的知名企业、产业联盟参与旅游项目投资和经营管理。全面启动南泥湾景区整体开发，加快推进黄帝文化园区、鲁艺文化产业园区、圣地河谷金延安二期、文安驿文化产业园区二期等文化旅游项目，全面开工建设黄河壶口文化产业园区、安塞黄土风情文化产业园区。大力发展科普教育、生态避暑、康体养生、冰雪运动等新型旅游业态。依托红色教育资源，打造特色品牌，发展红色教育培训产业，全年培训40万人次以上。持续抓好宜川、黄陵首批国家全域旅游示范县和延川、黄龙全省全域旅游示范县创建，推动梁家河、南沟等20个乡村旅游示范村提档升级。建立旅游导视系统和信息服务平台，发展智慧旅游。开展黄河壶口、乾坤湾、黄陵国家森林公园5A级景区创建，实现延安革命纪念馆、枣园、杨家岭、宝塔山、西北局5A级景区联合创建目标，提升景区整体形象和品牌影响力。启动甘谷驿古镇和延川乾坤湾、黄陵双龙市级文化旅游名镇建设。按照“季有活动、节有亮点”和“淡季不淡、旺季更旺”的要求，策划举办有影响力的文化旅游节会活动，有效提高游客吸引力。加强游客服务中心、旅游厕所、旅游标牌等基础设施建设，全要素提升旅游服务功能。高度重视具有延安特色的旅游纪念品开发，形成系列化、规模化、品牌化研发生产销售体系。持续开展旅游环境综合整治，运用新兴媒体，开展旅游精准营销，来延游客和旅游综合收入分别增长11.8%和14%。

（二）补齐四个发展短板，着力在培育经济增长新动能上下功夫

大力发展非能产业。支持延安高新区体制机制创新，将新区东区融入一体发展，力争晋升为国家级高新技术产业开发区。坚持创新引领、特色集聚、高端链接，打造新加坡中新膜科技园区、山东润峰新能源汽车园区等千亿级产业集群和英国生态科技园区、北航科技园区等百亿级产业集群，将延安高新区建设成为全市转型发展的技术支撑区、城市发展的产业支撑区。鼓励医药制造、高端装备、服装生产等企业加快发展，支持“延安卷烟”、“延安制药”等一批传统制造业品牌做大做强。制定出台加快建筑建材业发展意见，扶持一批建筑建材龙头企业发展壮大。坚持实施创新驱动战略，培育转型发展新动能，抓住证监会发挥资本市场作用服务国家脱贫攻坚战略的政策机遇，制定更加开放优惠的政策措施，吸引外地企业来延发展，推进与北京云真视讯、英雄互娱、898创新空间等企业合作，促进新经济加快发展。建立“市级统筹、县区招商、集中注册、成果共享”机制，打破行政区域限制，促进企业落地，培育区域经济增长新模式，让“飞地经济”在延安“破茧而出”。出台促进股权投资发展意见，设立产业投资基金和扶贫公益基金，吸引投资机构和社会资本投资。积极推进互联网与加工制造、现代农业、现代服务业渗透融合，培育消费经济、信息经济、分享经济等新业态。加快华为云计算数据中心、彩虹光伏玻璃、陕建新型材料等项目建设，推动战略性新兴产业发展。非能工业增加值占规上工业增加值比重达到13%。

培育发展新型服务业。落实服务业发展市场准入、用地保障、税费减免、融资担保、贷款贴息等支持政策，加快培育产权交易、评估服务、信息咨询等新型服务业，促进现代服务业多元发展。启动建设延安邮政快递物流双创产业园区，规范快递业、家政服务业服务标准。建设电子商务产业园区和市级众创空间，加快电子商务县级服务中心、村级服务站和农村物流配送体系建设，鼓励外贸企业开展跨境电子商务。加快推进能源装备、建材家居、农产品、小商品四大物流体系和延安现代综合物流园区、安塞物流产业园区等17个现代物流园区建设，启动新区农商城、上海亿丰小商品等大型市场综合体建设。支持利源物流发展城市零担货运和“冷链+电商”仓储配送。加强苹果、药品销售统计，促进交通运输、大宗商品批发等行业持续增长。完善商贸流通体系，健全商业网点，大力发展会展经济，释放城乡消费能力。支持延长石油集团建设成品油便利销售点。第三产业增加值增长10%。

全力支持非公经济发展。深化商事制度改革，坚持 “非禁即入”，放宽注册和准入条件，新增一批“四上”企业和小微企业。在项目策划、资金筹措、研发投入、设备购置、产品销售、税费减免等方面，支持民营企业发展。引导非公企业发行企业债和短期融资券，鼓励民间资本进入基础设施和公共服务领域。深入推进大众创业、万众创新，依托产业园区加快建设小微企业创业园、创业孵化基地，优先落实促进就业和创业担保贷款扶持政策。启动延大双创街区和新区双创社区建设。非公经济增加值占GDP比重提高到30%左右。

不断壮大县域经济实力。支持县区发挥资源优势和比较优势，加快发展新能源、新材料、新技术等新兴产业，改造提升传统产业，以经济结构的调整优化升级，培育富有县域特色的支柱产业和优势产业，打造规模较大、特色鲜明的区域板块经济，壮大县域经济综合实力，缩小与全省县域经济平均规模的差距。支持县域工业集中区申报省级高新技术产业开发区。坚持“有中生新、无中生有”，鼓励县区加强与中省智库、科研院校、大型企业合作，谋划论证、启动实施一批重大产业和基础设施项目，推动县域经济加快发展。

（三）发挥两大后发优势，着力在提升城乡一体化发展水平上下功夫

把2017年作为城乡一体发展提升年，充分发挥城镇化发展和基础设施建设空间大的后发优势，大力实施“基础设施十大建设工程”，增强城镇承载服务功能，提高发展保障能力。

提升城镇体系建设水平。狠抓城市修补和生态修复，采取针对性措施，补齐城市发展短板，完善城市功能，提高环境质量，塑造特色风貌，把好山好水好风光融入城市，建设天蓝、地绿、水净的美好家园，将延安打造成名副其实的“望得见山、看得见水、记得住乡愁”的宜居宜业宜游现代生态城市。坚持“富规划、穷建设”理念，完善提升老城和新区规划，实行刚性约束。加快推进新区尾留岩土工程和市政道路、供水供热排污等基础设施配套建设，建成新天地商业步行街等项目。用好棚改政策，继续推进老城改造，完成南门坡、宝塔山等棚户区改造。统筹实施地下综合管廊、智慧城市、海绵城市项目，加快城市道路环线建设，增强城市公共服务能力。推进公园广场、水体景观和城市绿化美化亮化，提升城市形象品位。深入推进“2+1”三城联创，出台城市市容环境管理条例，开展城市管理执法体制改革，实现全国文明城市和国家卫生城市、国家园林城市创建目标，国家节水型城市、环保模范城市创建力争完成省级验收。启动延安至安塞快速干道建设，加快推进延塞一体化进程。完善县城、重点镇基础设施和公共服务配套，提升城镇综合承载能力和人口聚集能力。城镇化率达到60%以上。

完善城乡基础设施条件。加快延安新机场、西延高铁、蒙华铁路、吴起至定边、绥德至延川、清涧至子长高速公路和县城过境、通村公路建设，开工建设子长至姚店、吴起至华池、延长至黄龙、黄龙至蒲城高速公路，建成志丹至南梁公路、沿黄公路，启动壶口通用机场建设。黄河引水工程实现向市区供水。加快陕北至关中和陕北至武汉电力外送通道建设。实施重点公共区域免费wifi覆盖工程。制定PPP项目实施优惠政策，至少向社会推介公布两批PPP项目，用好延安光大城市发展基金，撬动社会资本进入基础设施和公共服务领域，加快释放民间投资活力。打好“延安”金字招牌，紧跟中省政策走向，争取更多项目进入国家和省上盘子，确保项目接替有序、梯次推进。

（四）突破一个明显制约，着力在系统推进生态文明建设上下功夫

巩固生态建设成果。继续加强退耕还林、天然林保护、三北防护林等林业重点工程建设，抓好公路铁路沿线、河流两岸、重要景区、城镇社区等重点区域绿化，全年营造林50万亩。启动桥山、黄河国家公园建设。坚持封山禁牧，加强森林资源管护，巩固提升国家森林城市建设水平。认真实施国家山水林田湖生态保护修复项目，促进生态环境持续改善。完成治沟造地7.5万亩。治理水土流失面积1000平方公里。

突出重点领域环境治理。加强大气、水源、土壤等重点领域污染防治，下功夫解决油气生产和输送管线安全环保突出问题。坚持柔性治水，市域主要河流全面推行市县乡村四级河长制，加快延河综合治理，建设塘坝、涝池500座。多措并举治污降霾，紧盯油煤气生产、炼化、加工和供热、发电等重点企业，抓好PM2.5监测和煤改气、油改气、建筑渣土、扬尘治理，城区空气质量优良天数达到290天以上。

强化节能减排。突出抓好重点能耗企业落后产能淘汰和节能新技术、新工艺推广应用，坚决落实建设项目环境影响评价、防治污染措施“三同时”和企业排污许可、环境监测、环境信息公开、污染物排放总量控制等制度，加强城镇环保基础设施和工业污水、废气等治污设施建设，加大源头管控和监督执法力度，实现污染物达标排放。

改善农村人居环境。以净化美化农村为目标，以贫困村整体环境、贫困户生活环境改善为突破口，因村制宜、因户施策，全面开展农村人居环境综合大整治，彻底改变农村脏乱差状况。加大农村卫生改厕、生活垃圾和污水处理力度，统筹推进农村电网改造、道路畅通、安全饮水、危房改造工程，建设绿色家园。今年全市行政村全部建成清洁村，三分之一行政村建成生态村，10%以上行政村建成美丽宜居示范村。

（五）激发五大活力，着力在释放改革红利上下功夫

深化供给侧结构性改革。落实“三去一降一补”重点任务，支持煤矿企业兼并重组，引导退出2处煤矿45万吨产能。全面完成第二批40户国企改制任务。鼓励国有企业通过投资入股、联合投资、并购重组等多种方式，与非国有企业进行股权整合、资源整合、战略合作，大力发展混合所有制经济。把去库存与促进人口城镇化结合起来，构建租购并举住房制度，稳定房地产市场。清理规范中介服务和不合理收费，降低实体经济企业生产成本。

深化行政审批制度改革。深入推进“放管服”改革，认真落实“五证合一、一照一码”登记制度和“四张清单”制度，以政府权力的减法换取市场活力的加法，更好地为企业和群众创业创新松绑减负、清障搭台。实施“双随机一公开”监管机制，继续完善重大项目并联审批机制，全面提升行政审批效率。

深化财政金融改革。实行财政预决算公开全覆盖，全面推行绩效预算管理，做到花钱要问效、无效要问责。改变市对县转移支付投向，规范市对县补助，支持县区转型发展。清理财政配套政策，建立跨年平衡的滚动预算和资金调度机制，加大新增债券争取力度，保持财政平稳运行。转变融资观念，创新融资方式，拓宽融资渠道，优化资本运作，增强金融服务实体经济、推动转型发展的能力。

深化用地管理改革。严格控制建设用地规模，不断优化土地报批程序，积极探索工业用地租赁办法，保障重大产业、基础设施和民生项目用地需求。实行有保有压的差别化供地政策，深入开展闲置土地专项清理，盘活土地存量资源，提高城乡建设用地节约集约利用水平。用足用活城乡建设用地指标增减挂钩试点政策。落实农村土地所有权、承包权、经营权“三权分置”政策，加快推进农村集体产权制度改革，探索农村土地经营权、农民房屋权、林权抵押融资机制，建立风险缓释基金，实施农村产权交易试点。

深化对外开放。积极推进延榆一体化，主动融入大西安，加强与丝路沿线地区交流合作。培育壮大一批进出口企业，扩大进出口贸易规模。把招商引资作为推动转型发展的重要抓手，在北京、上海、深圳等经济发达城市增设“招商引资联络处”，紧跟国家政策导向，紧盯发达地区、行业龙头和知名民营企业，扎实做好项目论证，主动叩门招商，专项精准招商，着力引进一批带动行业发展、引领产业升级、补齐发展短板的大项目、好项目。强化招商引资硬考核、真问责，及时兑现优惠政策，确保招商质量、引资总量、到位资金、项目落地都有新突破。

（六）用心办好民生实事，着力在提高社会事业发展水平和增进群众福祉上下功夫

坚决打赢精准脱贫攻坚战。实现剩余1.02万贫困人口脱贫、224个贫困村脱贫退出和延川、延长、宜川3个国家级贫困县脱贫摘帽目标。完成易地搬迁集中安置点建设，按照缺什么补什么原则，完善水电路气讯网等基础设施，加快产业配套。加强贫困人口技能培训，坚持正确帮扶导向，激励贫困群众自强不息，坚决摒弃“靠着墙根晒太阳、等着别人送小康”的依赖思想。加强与无锡市扶贫协作，在产业转移、劳务输出、设施援建方面得到支持。争取中省网络扶贫试点落户延安。严格依据贫困户、贫困村和贫困县退出标准，实行第三方评估，保质保量完成脱贫任务。坚持脱贫攻坚期到村到户扶贫政策不变，持续巩固脱贫攻坚成果。

切实加强就业工作。继续实施促进创业带动就业五年行动计划，落实创业担保贷款、财政贴息和创办企业扣减税费政策，扩大就业渠道，鼓励引导高校毕业生自主创业、到非公企业就业。加强就业培训，促进高校毕业生、退役军人、城镇就业困难人员、去产能分流职工等重点人群就业，新增城镇就业1.5万人以上，城镇登记失业率控制在3.9%以内。

进一步提升社会保障水平。健全完善临时救助、医疗救助和城乡一体的特困人员供养制度，构建“救急难”、“大救助”新型社会救助体系。推进被征地农民参加城镇企业职工基本养老保险。加快发展社会化养老服务，全面推行居家养老社区服务模式。加快社会福利院和儿童福利院迁建，统筹推进助残、慈善、殡葬等事业发展，完善农村留守儿童关爱保护体系，做好特殊困难群体生活保障，筑牢困难群众基本生活防线。

有效扩大教育资源供给。持续改善城乡办学条件，加快薄弱学校改造、县城以上学校建设和义务教育学校标准化建设，统筹优化教育资源配置。实施第三期学前教育行动计划，提升学前教育水平。落实义务教育免试就近入学政策，严格划片招生，着力解决入园难和大班额、择校热等问题。强化教育教学视导，加强基层教师队伍建设，建立名优校长队伍和骨干教师体系，稳步提升教育教学质量。支持宜川中学建设区域性重点中学。支持延安职业技术学院创办特色鲜明的高职院校。

加强医疗服务保障。实施健康延安行动计划，健全重大疾病防控工作机制，优化公共卫生服务，倡导健康文明的生活方式，促进健康关口前移。启动综合医改试点工作，合理配置医疗资源，全面实施分级诊疗，方便群众看病。依托中国医科大学组建以市人民医院为龙头的第二医疗集团。推进综合医院、专科医院和9所社区卫生服务中心建设，加快市人民医院创三甲步伐，扶持和规范社会办医，全面提升基层卫生服务能力。理顺城乡居民医疗保险体制，推进医疗保险异地就医结算。加强妇幼健康服务，全面落实两孩政策。

推动文化科技体育事业加快发展。积极创建国家公共文化服务示范区，打造全国文化系统干部培训基地和陕北民歌、陕北说书、陕北道情传习基地，大力支持文艺精品创作。推进国家级陕北文化生态保护实验区建设，加强陕北民间艺术保护传承和创新发展。强化文化市场监管，全面净化文化市场。倡导全民阅读，建设文化延安、书香社会。注重家庭家教家风，弘扬社会主义核心价值观。启动延安博物馆、规划展览馆、石油博物馆建设。加强文物保护，完善博物馆体系，打造特色鲜明的中国革命博物馆城。推进科技研发中心和科技资源统筹中心建设，加快科技成果转化利用。健全完善人才引进、流动、激励机制，不拘一格用好人才。启动第十四届全运会延安场馆建设，广泛开展全民健身活动。

强化为民利民便民服务。实施城市巷道改造工程。加快城区停车设施建设。严格居民小区、商业建筑停车设施规划审批。优先发展公共交通，启动出租车行业改革。新建和改造便民市场，增加城市社区蔬菜直销亭、生鲜超市和城区早晚市布点，推进镇村放心粮油经销店建设。建立物业企业资质动态管理、社区评议等机制，加快智慧能源和老旧小区改造，提升物业管理服务水平。

推进社会治理创新。建立社区、社会组织、社会工作专业人才、社区志愿者“四社联动”工作机制，夯实社会治理基础，提高社区服务和管理能力。强化信访基层基础，积极排查化解矛盾纠纷，妥善解决突出信访问题。加快推进安全生产领域改革，强化道路交通、地质灾害、危险化学品等重点领域安全监管，提升应急防范和处置能力，坚决遏制重特大事故发生。坚持源头严防、过程严管、风险严控，开展省级食品安全示范县和乡镇示范所创建活动，保障群众舌尖上的安全。加强社会治安立体防控体系建设，全面提升治安、反恐和群体事件风险防控能力，严厉打击各类违法犯罪活动，不断提高人民群众的安全感和满意度。加强国防动员，支持部队建设和改革，扎实开展双拥工作，推进军民融合发展。加强民族宗教工作，促进民族团结。支持工会、共青团、妇联、工商联、科协、文联等群团组织工作。

各位代表，“人民对美好生活的向往就是我们的奋斗目标。”今年，我们将着力办好“人民幸福十件实事”：一是抓好21314户移民搬迁工程；二是实施57个城市棚户区改造和9个基础设施配套项目；三是在新老城区新建和改扩建幼儿园17所、小学14所、初中9所，加快延安大学新校区建设；四是完成市中医院迁建，加快精神病医院建设；五是加快新区博物馆片区、中国革命文艺家博物院建设；六是建设10个城区休闲广场和山体公园；七是建设10个城区停车场（楼）；八是建设10个城区便民市场；九是完成城区20条巷道改造；十是提升市区7个社区服务功能。

四、加强政府自身建设

人民满意是政府工作的最高标准。牢固树立“四个意识”，把对党绝对忠诚作为干事创业的灵魂，坚定不移落实市委的决策部署，切实提高执行力、创新力和公信力，努力建设人民满意的服务型政府。

解放思想，勇于责任担当。跳出“沟道思维”，站在“宝塔之巅”看延安，深化市情认识，直面困难矛盾，冲破思维定势、工作惯性和路径依赖，以开放的思维和全球的视野谋划发展。打破体制机制障碍，突破利益固化藩篱，以逢山开路、遇水架桥的勇气，以功成不必在我的境界，强化责任意识、忧患意识和赶超意识，敢于担当，锐意改革，善于用市场手段破解难题，用法治思维推动工作，在裁弯取直中加快发展，彻底摆脱经济增速长期在全省垫底的被动局面。

依法行政，规范权力运行。把法治理念贯穿政府工作全过程，严格按照法定权限和程序用权履责。健全科学民主决策机制，完善依法行政制度体系，严格规范公正文明执法，努力做到法无授权不可为、法定职责必须为，不越位、不缺位、不错位、能补位。依法接受市人大及其常委会的法律监督和工作监督，主动接受市政协民主监督，认真听取各民主党派、工商联和无党派人士意见，提高人大代表建议意见和政协委员提案办理质量。深入开展“七五”普法，落实“谁执法谁普法”责任，增强全社会普法学法守法用法观念。

改进作风，矢志苦干实干。认真落实中央八项规定精神和国务院“约法三章”，严防“四风”问题反弹。坚持政府过“紧日子”、让群众和企业过“好日子”，持续降低行政运行成本，“三公”经费只减不增。坚持求真务实，听实话、察实情、出实招、求实效。大力发扬“钉钉子”精神，让真抓实干成为政府工作人员的自觉追求，努力用干部的辛苦指数换取人民的幸福指数。进一步精简会议文件，减少程序性运行，聚精会神谋发展、解难题、抓落实。坚持重点工作月通报、季讲评、观摩评议、督查约谈制度，继续办好“电视问政”。坚持正向激励、反向倒逼，严格落实“三项机制”，真正使想干事的有机会、会干事的有舞台、能干事的有地位，让不作为、慢作为、乱作为无藏身之地。

高效服务，优化发展环境。牢固树立“马上就办、办就办好”理念，以保障企业生产、项目建设、引资落地为重点，持续开展投资环境集中整治，以零容忍的态度、猛药去疴的决心，坚决向故意设卡、刁难设阻、强买强卖的人和事亮剑，严厉打击破坏投资环境的村霸和宗族势力。严格落实执法人员持证上岗和行政执法责任制，提高执法效率和规范化水平。实行群众办事首问负责、限时办结和清单回执制度，推进“互联网+”政务服务，再造流程，实现一号申请、一窗受理、一网通办，让信息数据多跑路，让群众和企业办事少走冤枉路、心里不添堵，让革命圣地始终充满公平正义的正能量。

清正廉洁，严守纪律规矩。认真贯彻党的十八届六中全会精神，严格执行《准则》和《条例》，敬畏权力，严守底线，切实提高政府工作人员自我净化、自我完善、自我约束能力。加强对重点领域、关键环节、重要岗位权力运行的监督制约，坚持用制度管权、管事、管人，推动公共资金、国有资产、领导干部经济责任审计全覆盖。严格落实党风廉政建设主体责任，构建“亲”、“清”新型政商关系，惩治群众身边的“微腐败”。强化行政监察，严肃查处以权谋私、失职渎职和贪污腐败行为，以实际行动树立为民务实清廉政府形象。

各位代表，全面小康时不我待，美好愿景令人向往。让我们更加紧密地团结在以习近平同志为核心的党中央周围，认真贯彻落实省委、省政府的决策部署，在市委的坚强领导下，低调务实不张扬，撸起袖子加油干，凝心聚力，追赶超越，为延安人民生活得更幸福更美好而努力奋斗！
